# Wear-Resistant TiC Strengthening CoCrNi-Based High-Entropy Alloy Composite

**DOI:** 10.3390/ma14164665

**Published:** 2021-08-19

**Authors:** Yanlin Cai, Yonggang Tong, Yongle Hu, Hongfeng Huang, Xiancheng Zhang, Manyu Hua, Shan Xu, Yongbing Mei, Chengbiao Ma, Zhifeng Li

**Affiliations:** 1 College of Automobile and Mechanical Engineering, Changsha University of Science and Technology, Changsha 410114, China; 15886627187@163.com (Y.C.); huamanyu20210804@163.com (M.H.); 2 Key Laboratory of New Processing Technology for Nonferrous Metal & Materials Ministry of Education, Guilin University of Technology, Guilin 541004, China; hhfeng@glut.edu.cn; 3 School of Mechanical and Power Engineering, East China University of Science and Technology, Shanghai 200237, China; xczhang@ecust.edu.cn; 4 China Railway Construction Heavy Industry Corporation Limited, Changsha 410100, China; xushan@crchi.com (S.X.); meiyongbing@crchi.com (Y.M.); machengbiao@crchi.com (C.M.); lizhifeng@crchi.com (Z.L.)

**Keywords:** high-entropy alloy, microstructure, TiC, wear resistance, mechanical properties

## Abstract

In order to improve the wear resistance of CoCrNi alloy, TiC was introduced into the alloy and wear-resistant CoCrNi/(TiC)_x_ composites were designed. The effects of TiC contents on the microstructure, mechanical properties, and wear resistance of CoCrNi matrix were investigated, respectively. It was found that the TiC produced dissolution and precipitation process in CoCrNi alloy, and a large number of needled and blocky TiC particles were precipitated in the composites. The compressive yield strength of CoCrNi/(TiC)_x_ composites increased with the increasing TiC content. Compared with the CoCrNi alloy, the yield strength of CoCrNi/(TiC)_x_ composites increased from 108 to 1371 MPa, and the corresponding strengthening mechanism contributed to the second phase strengthening. The wear resistance of CoCrNi/(TiC)_x_ composites was also greatly improved due to the strengthening of TiC. Compared with the CoCrNi alloy, the specific wear rate of CoCrNi/(TiC)_1.0_ alloy was reduced by about 77%. The wear resistance of CoCrNi/(TiC)_x_ composites was enhanced with the increasing content of TiC addition.

## 1. Introduction

Remanufacturing is one of the most efficient ways of recycling worn parts since it consumes only a fraction of the energy, cost, and material required for new parts. The remanufacturing product meeting or exceeding the performance of new products, significantly reduces the adverse impact on the environment [1,2]. Wear failure is one of the main failure styles of mechanical parts [3,4]. The development of wear-resistant surfaces is beneficial to improve the life of construction machinery, improve equipment reliability and save resources. To obtain a surface with excellent wear resistance, it is necessary to develop new and wear-resistant materials.

Traditional wear-resistant materials include gray cast iron, copper alloys, bearing steel, etc. The development of high-entropy alloys in recent years has provided new ideas for the design of high-performance materials. The emergence of high-entropy alloys has broken the traditional alloy design ideas. The formation of simple FCC, BCC or HCP solid solutions is promoted due to the high configuration entropy of high-entropy alloy. It has a series of excellent properties such as high strength, good wear resistance, corrosion resistance, and high-temperature oxidation resistance [5,6,7,8,9]. Among these alloys, CoCrNi alloy with a single-phase FCC structure has attracted much attention due to its excellent ductility, work hardening, and corrosion resistance [10,11]. However, the tensile strength of CoCrNi alloy does not exceed 700 MPa, and the hardness is less than 400 HV [12,13]. The lower strength and hardness make the wear resistance of CoCrNi alloy to be improved.

Introduction of ceramic particles is an effective way to improve the wear resistance of the alloys [14,15,16,17,18,19]. For example, the addition of TiB_2_ in the matrix of (AlCrFeMnV)_90_Bi_10_ high-entropy alloy reduced the wear rate by 85%, which significantly improved the wear resistance of the material [20]. TiC is often used as a reinforcing agent due to its high hardness, high melting point, good wettability, and chemical stability [21,22,23,24,25,26,27,28]. Adding TiC into the CoCrNi alloy to form a CoCrNi/TiC composite will play the advantages of CoCrNi and TiC, and is expected to form a new type of wear-resistant material. However, there is no relevant report on the improvement of wear resistance by adding TiC into CoCrNi alloys. The purpose of this investigation is to produce a highly wear-resistant CoCrNi/TiC composite, and this idea was verified by experiments. The effects of TiC content on microstructure, mechanical properties, and wear resistance of CoCrNi alloy were investigated.

## 2. Materials and Methods

CoCrNi/(TiC)_x_ (x = 0.6, 0.8, 1.0; x is the molar ratio) composites were designed by adding a non-equal mole ratio TiC into the CoCrNi medium entropy alloy. The raw materials used were small pieces of Co, Cr, and Ni with a purity of 99.5%. TiC powder with 99.5% purity was introduced into the CoCrNi alloy, and TiC powder is supplied by the Chengdu Huayin Powder Technology Company Limited. The alloys were prepared by a conventional, vacuum, non-consumable arc melting method. To increase compositional homogeneity, the ingot was inverted and remelted five times. The samples with different molar ratios were marked as samples T0 (CoCrNi), T6 (CoCrNi/(TiC)_0.6_), T8 (CoCrNi/(TiC)_0.8_), and T10 (CoCrNi/(TiC)_1.0_), respectively.

**Figure 1:**
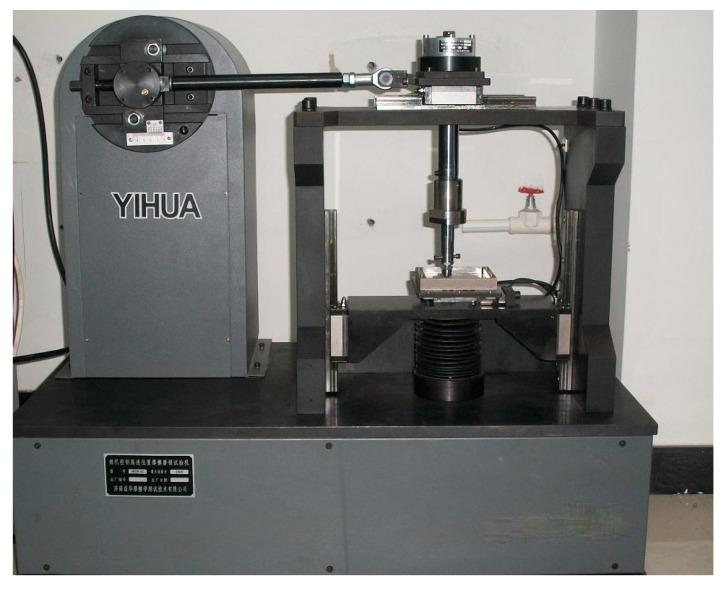
MDW-05 reciprocating friction and wear tester.

X-ray diffractometry (XRD, Cu Kα radiation, Rigaku D/Max 2550VB, BRUKER D8 Advance X-ray Diffractometer, BRUKER, Germany) was used to analyze the phases in the composites. The scanning range was 20–80°, and the scanning speed was 5°/min. Scanning electron microscopy (SEM) was used to characterize the microstructure, and energy-dispersive spectrometry (EDS) was used for element composition analysis. Samples with the size of Φ5 × 8 mm were cut from the ingots, and a CMT5105GL electronic universal tensile testing machine was used to test the compressive mechanical properties at room temperature with a loading rate of 0.5 mm/min (without lubrication). The MDW-05 reciprocating friction and wear tester (Yihua Tribological Testing Technology Company Limited, Jinan, China) were used to test the anti-friction and wear properties of the alloy (as shown in Figure 1). The grinding ball was an Al_2_O_3_ ball with 6 mm diameter. The experimental load is 50 N and the loading length is 10 mm. The running time was 30 min, and the running frequency was 2 Hz. The dry sliding wear test was carried out at room temperature without lubrication. The samples were washed with acetone ultrasonic before and after wear. After the friction experiment, the width and depth of the wear scar were measured by a two-dimensional profiler (Shenzhen Zhongtu Instrument Company Limited, Shenzhen, China). Observation of the grinding ball wear surface was done by an optical microscope.

## 3. Results and Discussion

### 3.1. XRD Analysis

**Figure 2:**
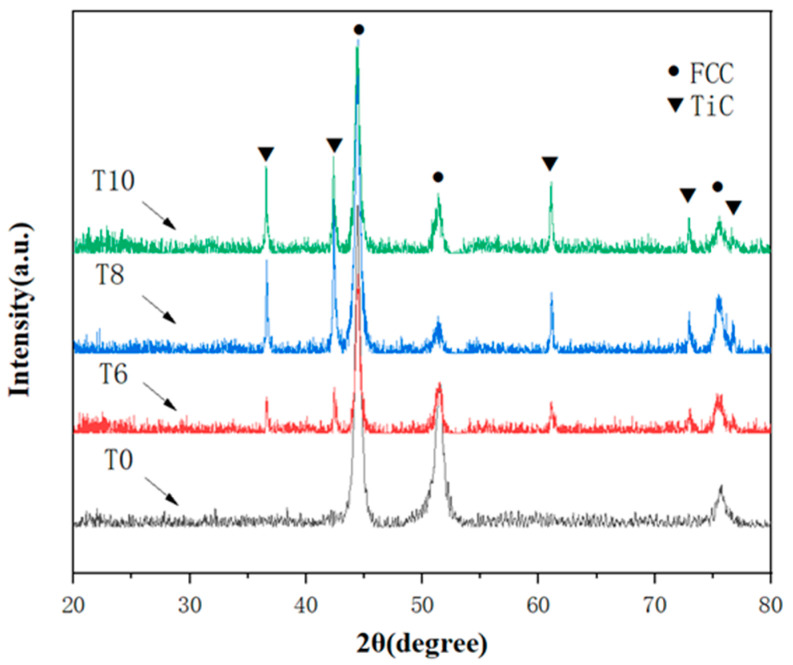
XRD patterns of CoCrNi/(TiC)_x_ (x = 0, 0.6, 0.8, 1.0) composites.

XRD patterns of CoCrNi/(TiC)_x_ (x = 0, 0.6, 0.8, 1.0) composites are shown in Figure 2. The results show that the sample T0 is a single-phase FCC structure. TiC diffraction peaks were found in T6, T8, and T10 samples in addition to the FCC phase. With the increase of TiC content, the TiC diffraction peak gradually strengthened, indicating that the TiC phase increased in the composites. In addition, no other complex intermetallic compounds were found in the composites. This suggests that the structure of the composites is the same as we expected.

### 3.2. Microstructure

**Figure 3:**
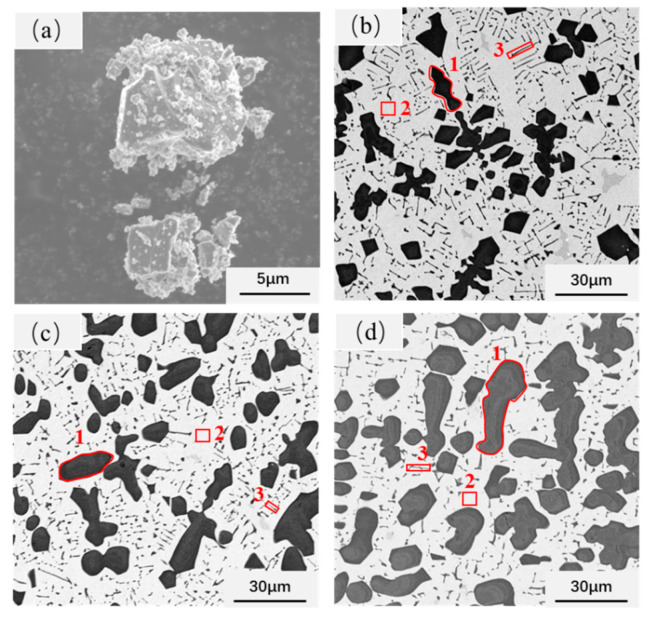
SEM images of CoCrNi/(TiC)_x_ (x = 0.6, 0.8, 1.0) composites and TiC powder: (**a**) TiC powder; (**b**) CoCrNi/(TiC)_0.6_; (**c**) CoCrNi/(TiC)_0.8_; (**d**) CoCrNi/(TiC)_1.0_; The block TiC are region 1, the dendrite core are region 2, and the interdendrite are region 3.

**Figure 4:**
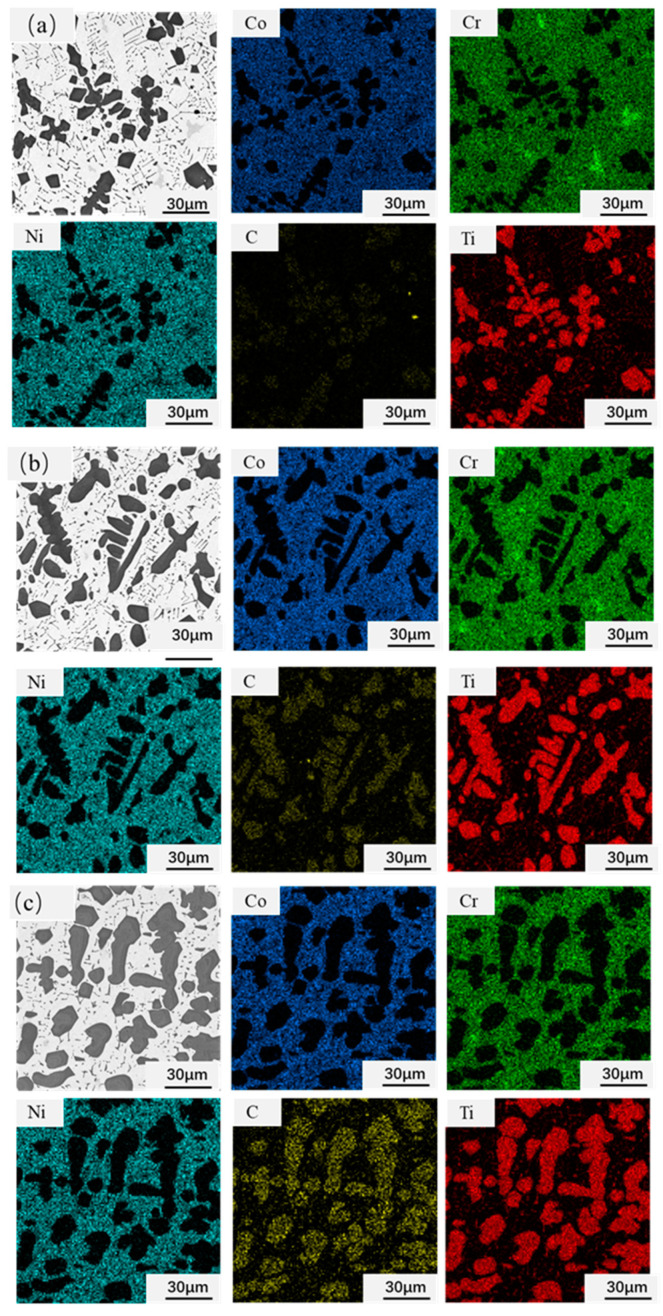
Elemental maps of CoCrNi/(TiC)_x_ (x = 0.6, 0.8, 1.0) composites: (**a**) CoCrNi/(TiC)_0.6_; (**b**) CoCrNi/(TiC)_0.8_; (**c**) CoCrNi/(TiC)_1.0_.

Figure 3a is the morphology of the original TiC powders. The figure shows that the original TiC particles are irregular blocks with an average size of less than 10 μm. The microstructure of CoCrNi/(TiC)_x_ (x = 0.6, 0.8, 1.0) composites are shown in Figure 3b–d. It can be seen that the composites are composed of three phases. Combined with the point scanning of the energy spectrum (Table 1) and surface scanning of the energy spectrum (Figure 4), it can determine that the white microstructure is the CoCrNi matrix, and the black blocky and needled structures are TiC particles. Compared with the initial TiC particles, it can be concluded that the appearance of the needle-shaped TiC precipitates in the composite. This indicates that TiC produced dissolution and precipitation processes in the CoCrNi alloy. The width of the needle TiC was measured to be no more than 1 μm, and the length varied from a few microns to more than a dozen microns. However, as the TiC content increased in the composites, the precipitation of a large number of blocky TiC particles decreased the volume fraction of the needled precipitates. The size of bulk TiC in the composites is proportional to the TiC content (Figure 3d). Additionally, the average size of the bulk precipitates grew from about ten microns to tens of microns, and there is a tendency to connect with each other. Moreover, a small amount of C and Ti elements were detected in the CoCrNi matrix (Table 1), indicating that a small amount of TiC was dissolved in the CoCrNi matrix.

### 3.3. Mechanical Properties

**Figure 5:**
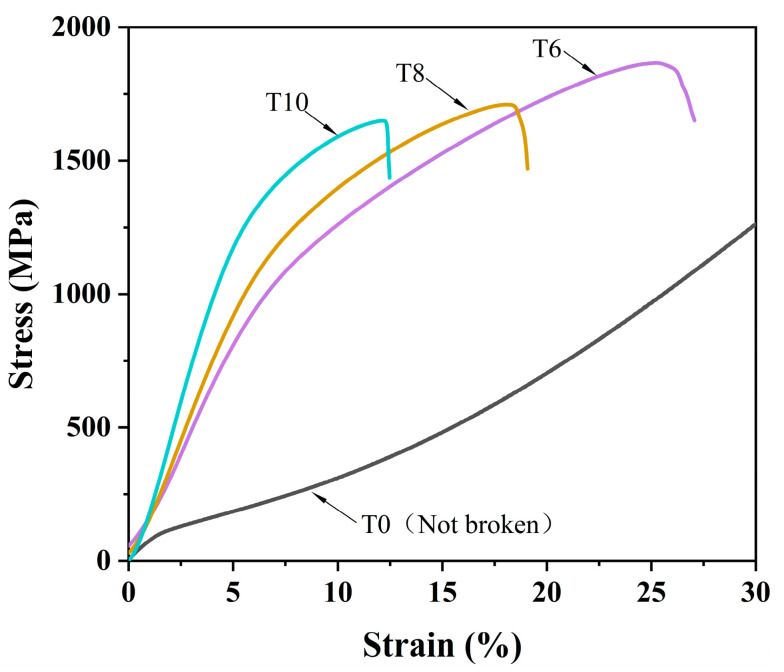
Typical engineering stress-strain curves of CoCrNi/(TiC)_x_ (x = 0, 0.6, 0.8, 1.0) composites.

The compressive mechanical properties of CoCrNi/(TiC)_x_ (x = 0, 0.6, 0.8, 1.0) composites were measured and shown in Figure 5 and Table 2. It can be seen from the figure that the introduction of the TiC ceramic phase significantly enhanced the yield strength of the composites. Compared with the yield strength of CoCrNi alloy at 108 MPa, the yield strength of samples T6, T8, and T10 increased to 883, 1102, and 1371 MPa, respectively. The increase of yield strength can be attributed to the second phase strengthening generated by TiC particles in the composites. However, the increase of TiC content led to a simultaneous decrease in the compressive strength and plasticity of the composites. Such a result is related to the microstructure change of the composites. The size of the block TiC particles that precipitated from the composites with high content of TiC addition tends to increase and connect onto pieces (Figure 3b–d), which may decrease the composite’s deformation capacity.

**Figure 6:**
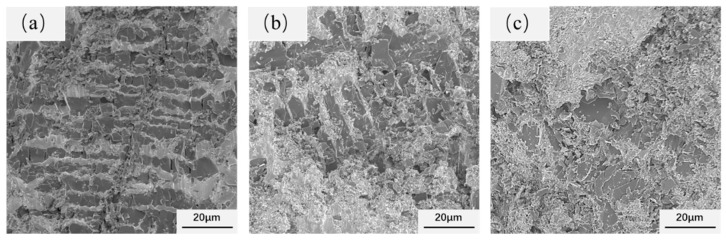
Fracture surfaces of CoCrNi/(TiC)_x_ (x = 0.6, 0.8, 1.0) composites: (**a**) CoCrNi/(TiC)_0.6_; (**b**) CoCrNi/(TiC)_0.8_; (**c**) CoCrNi/(TiC)_1.0_.

The fracture morphologies of CoCrNi/(TiC)_x_ (x = 0.6, 0.8, 1.0) composites are shown in Figure 6. The CoCrNi alloy was pressed into a cake shape without cracks, which confirms that the ductility of CoCrNi alloy is very good. However, the addition of TiC changed the fracture mode of composites from toughness to brittle fracture. With the increase of TiC content, the fracture mode of the composites did not change significantly and remained dominated by the dissociative fracture. In addition, the increase in TiC content led to a further increase in the size of the bulk TiC particles, and the fracture of the TiC particles was the main fracture site (Figure 6). This means that the deformation of the matrix will transfer the load to the blocky TiC particles. The fracture mode of this type of composites is reported to depend on the relative strength of the interface and the reinforcing particles [29,30]. The particle fracture occurred in this work, indicating the high interfacial strength of the composites.

### 3.4. Wear Properties

**Figure 7:**
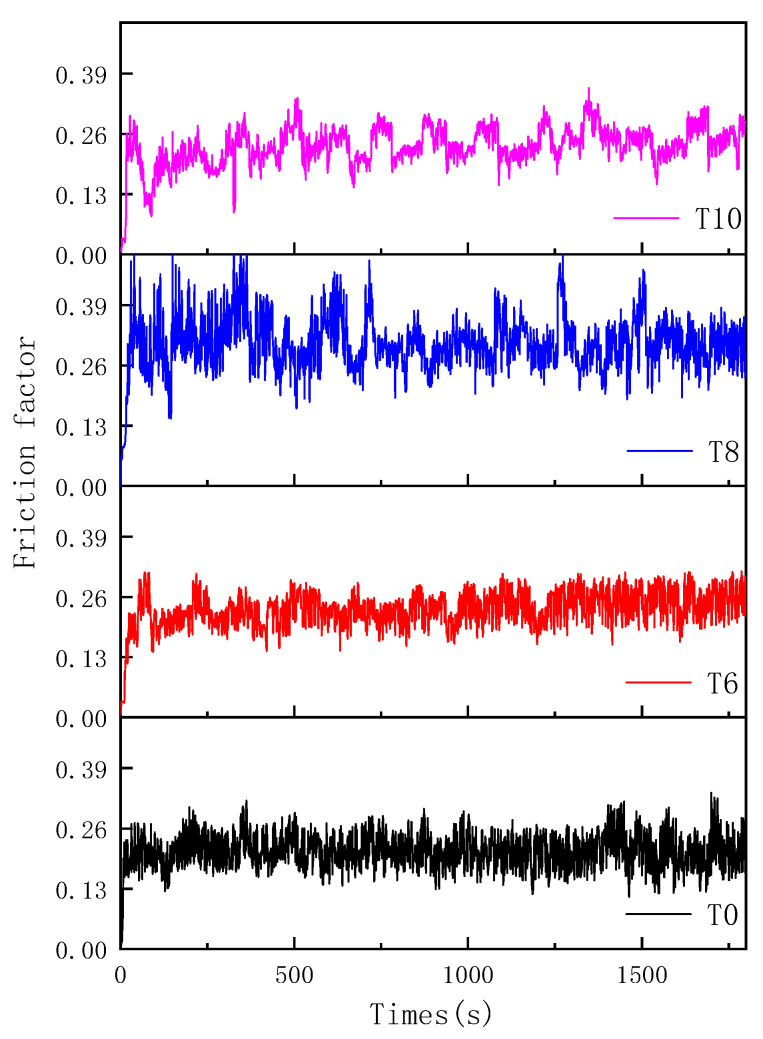
Friction coefficient of CoCrNi/(TiC)_x_ (x = 0, 0.6, 0.8, 1.0) composites.

**Figure 8:**
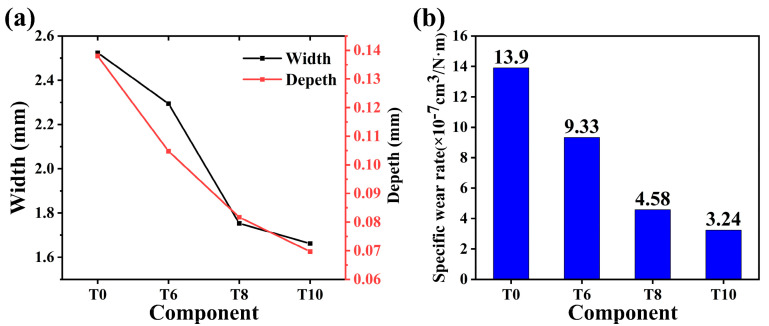
(**a**) Wear width-depth diagram of CoCrNi/(TiC)_x_ (x = 0, 0.6, 0.8, 1.0) composites; (**b**) the specific wear rate diagram of CoCrNi/(TiC)_x_ (x = 0, 0.6, 0.8, 1.0) composites.

**Figure 9:**
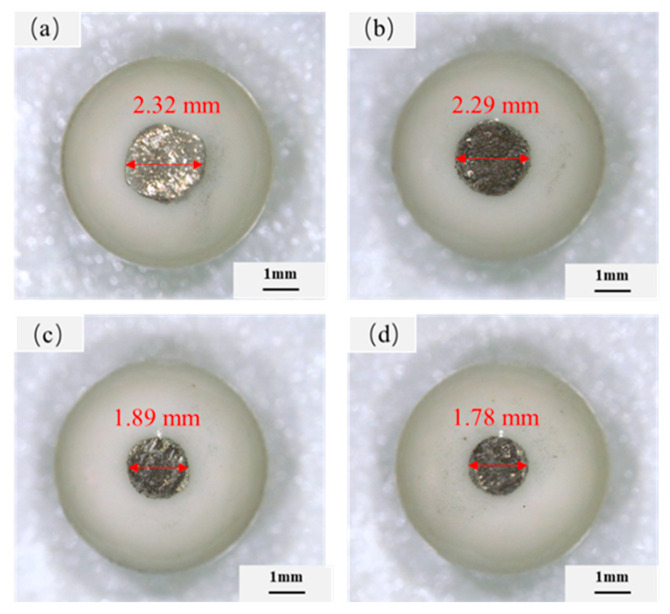
Optical images of Al_2_O_3_ counter face used in wear tests for the CoCrNi/(TiC)_x_ (x = 0, 0.6, 0.8, 1.0) composites: (**a**) CoCrNi; (**b**) CoCrNi/(TiC)_0.6_; (**c**) CoCrNi/(TiC)_0.8_; (**d**) CoCrNi/(TiC)_1.0_.

Figure 7 is the friction coefficient curve of CoCrNi/(TiC)_x_ (x = 0, 0.6, 0.8, 1.0) composites under 50 N load. It can be seen from the figure that the friction coefficient of the composites increases rapidly at the initial stage of friction. The friction coefficient tends to be stable in less than 2 min. With the increase of TiC content, the friction coefficients increased firstly and then decreased, and the average friction coefficients of T0, T6, T8, and T10 were 0.21, 0.23, 0.31, and 0.23, respectively. In order to analyze the wear of CoCrNi/(TiC)_x_ alloys quantitatively further, the width and depth of the wear scar of the samples were measured by a profiler, as shown in Figure 8a. It was found that under the same conditions, the width and depth of the samples wear scar decreased with the increase of TiC content. Among them, the grinding depth and width of sample T10 were the smallest. It indicates that the wear resistance of the composites increases with the increase of TiC content, and the sample T10 has the best wear resistance. In addition, the wear condition of the grinding balls was also analyzed, and the optical microscopic results are shown in Figure 9. The wear cross-sections of samples T0, T6, T8, and T10 composites against the grinding balls are all circular cross-sections. The wear diameter of the ball decreases with the increase of TiC content, from 2.32 to 1.78 mm. The change rule of wear diameter of grinding ball is consistent with the result of wear scar width. Moreover, Figure 8b is the calculated specific wear rate diagram of CoCrNi/(TiC)_x_ (x = 0, 0.6, 0.8, 1.0) composites. The specific wear rate is the volume loss per unit meter per unit load. It can be seen from the figure that the specific wear rates of samples T0, T6, T8, and T10 were about 1.39 × 10^−6^, 9.33 × 10^−7^, 4.58 × 10^−7^, and 3.24 × 10^−7^ cm^3^/(N·m), respectively. The specific wear rates of the composites gradually decrease with the increase of TiC content. Compared with the CoCrNi matrix, the specific wear rate of sample T10 was reduced by nearly 77%, which again proves that the increase of TiC content improves the wear resistance of the composites. The improvement of wear resistance of composites should be resulted from the precipitation of a large number of TiC ceramic particles in the microstructure.

## 4. Conclusions

In conclusion, wear-resistant CoCrNi/(TiC)_x_ (x = 0, 0.6, 0.8, 1.0) composites were prepared, and the microstructure, mechanical properties, and wear resistance of the composites were investigated. The key results of this work were as follows:

(1) TiC produced a dissolution and precipitation process in CoCrNi alloy, and the alloy’s structure transformed from single-phase FCC to FCC + TiC dual phases.
(2) The compressive yield strength of the CoCrNi/(TiC)_x_ composites was proportional to the TiC content, and the yield strength of the composites was increased to 1371 MPa. The increase of strength was mainly attributed to the second phase strengthening.
(3) The wear resistance of CoCrNi/(TiC)_x_ composites increased with the increasing TiC content. Among them, the specific wear rate of CoCrNi/(TiC)_1.0_ alloy was the lowest, which was nearly 77% lower than the CoCrNi matrix.

## Figures and Tables

**Table 1:** EDS analysis results of typical micro zones indicated in Figure 3, in atomic percentage, %.

Position	Co	Cr	Ni	Ti	C
Entire area of Figure 3b	18.14	17.10	17.96	14.69	32.11
Figure 3b–1	–	–	–	47.51	52.49
Figure 3b–2	28.85	22.04	27.97	2.16	18.98
Figure 3b–3	12.52	11.79	11.53	15.51	48.65
Entire area of Figure 3c	15.65	14.78	15.39	18.34	35.84
Figure 3c–1	–	–	–	47.51	52.49
Figure 3c–2	28.66	22.06	27.75	2.46	19.07
Figure 3c–3	15.09	17.04	15.70	19.46	32.71
Entire area of Figure 3d	12.25	11.88	12.22	23.06	40.59
Figure 3d–1	–	–	–	48.12	51.88
Figure 3d–2	28.02	23.99	27.58	2.65	17.76
Figure 3d–3	21.40	19.44	21.77	5.95	31.44

**Table 2:** Compressive mechanical properties of CoCrNi/(TiC)_x_ (x = 0, 0.6, 0.8, 1.0) composites.

Alloying Constituent	Yield Strength (MPa)	Compressive Strength (MPa)	Fracture Strain (%)
T0	108	–	–
T6	883	1865	25
T8	1102	1709	18
T10	1371	1649	12

## Data Availability

The data presented in this study are available on request from the corresponding author.
